# Supplementary material for: Identification of Shoot Differentiation-Related Genes in Populus euphratica Oliv
Source: Genes (Basel). 2019 Dec 11;10(12):1034. doi: 10.3390/genes10121034 (PMC6947848; doi:10.3390/genes10121034)
Supplement: Supplementary file 1 [file genes-10-01034-s001.zip › Supplementary Materials 1/Appendix.docx]

Appendix 11 sequences related to buds differentiation genes

> LOC105115136

ATGAGTATTAATAACAATGGAAGTAATAATAACTGGTTAGGCTTCTCTCTTTCGCCCCATATGAAAATGGAGGCTGCTTCTGAATCCCAACATCATCATCAGTACCATCATCAAACCCAAACCCAAACTCATGCTCCTGCTCCTGCTGCTGATTCTGTTTCTGCTGCCGTTCCAACAAGTTTCTATCTTTCTACTTCTCTCTTCAACAGTTCTGGAATCTGTTTTGGAGTTGGAGAAAATGGTGGCTTTCACTCTCCTTTGTCTGTTATGCCCCTCAAATCTGACGGCTCTCTTTGTATCATGGAAGCTCTCACTAGATCACAGCCTGAAGGAATGGTGTCGAGTCTATCACCAAAACTGGAGGACTTCCTAGGTGGTGCAACCATGGAAAGTCATCATTACAGTAGCCATGAAAGGGAAGCCATGGCCTTAAGTCTAGACAGCATATATTACCAGCAAAGCTCAGAGCCAGAAACTAATGGACAACATTCTCTCAACCTTCATGAACCATATAGGCAGCAAGACCAACAGTTTTCAGTTCAAACCCACCCATATTATTCTGGAATAGCATGCCAAGGATTGTATCAGGCCCCACTGGAGGGAGAAACCAAGGGTACTCAGCTTGCAGATTGTAATTCACCTATTCCTCTAATGGGAGACGATGAGCTGCCTCGCTTGAAACACTGGGTTGCTAGACACTATTCCTCGCCAAATGCACTGGAGCAGCAGATTAATAGTGGCATGGTTAATGATAGTGGGGCTTCTTGTTCTGCTAGTGCTGTAGGTTGTGGGGATTTACAGTCTCTCAGCTTGTCTATGAGCCCTGGTTCGCAGTCAAGCTGCATTACAGCTCCAAGGCAGATCTCACCTGCTGGCACTGAGTGTGTGGCCATAGAAACAAAGAAGCGAGGCCCTGTAAAAGGGGGTCAAAAACAGCCTGTTCATAGGAAGTCTATTGACACATTTGGCCAAAGAACATCACAGTATAGAGGCGTTACAAGACACAGATGGACTGGTAGATATGAAGCCCATCTCTGGGACAATAGTTGTAAGAAGGAAGGCCAGACCAGGAAAGGAAGGCAAGTTTATCTGGGGGGTTATGATATGGAAGAGAAAGCTGCAAGGGCTTATGATCTTGCTGCCCTTAAGTACTGGGGACCTTCAACTCATATAAATTTTCCGTTAGAAAATTACCAGGAAGAACTTGAAGAAATGAAAAACATGGGCCGCCAGGAATATGTTGCACATCTACGGAGAAAAAGTAGTGGGTTTTCTAGGGGGGCCTCAATCTACAGAGGAGTAACAAGGCATCACCAGCATGGAAGATGGCAAGCAAGGATAGGCAGGGTTGCAGGAAACAAGGACCTTTATCTTGGGACGTTCAGCACCCAAGAGGAAGCTGCTGAAGCTTATGATATTGCTGCGATCAAATTTCGGGGGGTTAATGCTGTGACAAACTTTAACATAACAAGATATGATGTTGATAGGATCATGGCCAGCAACACGCTTCTAGCTGGAGAACTAGCCAGGCGAACCCGAGACACAGAATCTAGCATTGAGGCCATTGACTATAACACATCAACACAGAACAATTGTGTTTTATACCAATCTCCGCAGGAACAGCCAAATGCTAGTGGAGAATCTCTTGATCAGAAGTCAATGAGTGCTGGGAATTATCGAAGCACCTCTTTCTCAGTGGCAGTGCAAGATCTTATGGGTATGGATCATTCAGTGAACTCTAGCCAGCTAGAGGTAGATGAATCAGCCAAGCTAGGAACTCATCTCTCGAACCCCTCATCTTTGGTGACCAGTTTGAGCAGCAGCTCTAGAGAAGCTAGCCCTGACAAGACTGGAACCCCTGTGCTCTTTGCTAAACCTCCACGGGCATCAAAGTTCATAGGCCCTACAACCAGCGTTACCCCTTGGATCCAAGCAGCAGCAGCCCAATTGAGGCCAGCAGGAATCTCCATGGCTCACTTGCCAGTTTTTGCTGCATGGAATGATACCTAA

>LOC105112164

ATGCTTATACAGCTCAGGCTGACAACGGAACAAGACACAAGAACAAGAACAAGTATGGAGGAACTCCCTCTAGGTTACCGCTTCTACCCAACAGAAGAAGAGCTGATCTCCTTCTACCTGCATAATAAGCTGGGAGGGGGGAGGCAAGAAAGTCTACAACGTGTTATTCCAGACATCAGCATTTACGACATCGAACCATGGGATCTTCCAAAGCTTTCAGGAGAACTATGTCAGGCAGACACCGAGCAGTGGTTCTTCTTTACACCCAGGCAAGAGAGAGAAGCTAGGGGAGGGAGACCTAACCGGACTACAGCATCCGGGTACTGGAAGGCCACAGGCTCTCCTGGTTATGTTTACTCGTCGGACAATCGTGTGATTGGATTGAAGAAAACCATGGTGTTCTATACGGGAAAAGCTCCAAGTGGAAGAAAAACCAAATGGAAGATGAATGAGTACAGAGCCATTGAAGTTCACGAATCTTCTAGCAATGCTACTCCAAAGTTGAGGCATGAATTCAGCTTGTGTCGAGTCTATGTGGTATCAGGAAGCTTTCGTGCATTTGATAGGCGGCCACTAGAAGCTGTGACAAGAGAGACGCAGCTTCATGGAGAGGGAGCTACAACATCTGCTCGGGGCCCTGATCCAACTGTGGATATGACAAGCTCACCTGAAACGTCATACTCATTGGAAGACCATGTTGATCACCCCTGGACTGCAGCAAGTGCCAACTGGGGGGCAGTAGATGGTCTAGAACCACCTGCATGGGAATGGCCAGGACAACTGGATTGGCCCTGA

>LOC105121805

ATGGCAGCAGCGGCCTCGTATTTACTCGGTAGACTTAGCAACGACAACATTAGTATCAATAACATCACCATTGATAATAGCGGTGGCAGTTGCAGCAACATCGACAACGCCAGTACTGCTTCATTGACAAGTGCCTCCTTGCTTACCAACGGCGGTAACCAAGCAACGACAATACCAGTAGTAGAAGCAGCAACTGGAAGGATGGTGAGAAAGAGGATGGCTTCAGAGATGATGGAAGTGCAATCAACAATGACTCCTCTAAATCACAGGTTATCTCGTGGTAACACCAACGTCTATTCACTTTCAATATCAGACAATAATAATGATGATGTTATTGGTATGACTAGAGGTATCGGTGCCTCCTCTTTTACTAGTTGTTCTAACAACAATATTAACCCTAATAACCTCAACCCAATCTTATATCCACCCCTCAACTACTCCACTATGACTTCTATGTTACCTTCTTCCACGAACTTGACAGCTATTACATCAGCCGGGTCTGCTTCTGTGTCCGTTTCTGGGTTTTTATCCTCTACGCCATCGACCAACTTAGCCACTATCTGTAATGACAACCAATCCCAACTCCCAGCCGTATGTGGTTTCTCTGGTCTGCCCTTGTTCCCTCCTGAAAGAGAAAGAAATATTGTCCGTTCGAACGCCGTCCCTCCACCCGGCCTTATCACTACCAGCTCTGCTTCTACCCCTACGCCCCCTATGGAAGATGCCGCACCCGCCACTGCCTGGATTGATGGCATCATAAAGGACCTGCTCCACAGTTCCACCAACGTGTCCGTTCCTCAGCTCATTCAGAATGTGAGGGAGATTATCTACCCTTGCAACCCCAATCTAGCCTCCCTTCTCGAGTACAGGCTCCGGTCCCTGACAGACCCGATAATCCCGGCCAATATTTACCCGGTGGAGAGGAGACGGAATAAGGAGGCAGCAGCAGTGCCATTGCCGTTTCAGAGGAATTATAATCAAGGTCATGCCCCTTCCGGCCTCTCTCTTGATCTTGATCATGTCTCCAATTCCGCTCCTCCAGTTTCATCACATGTTGTAAGTCACTACTCTAACTGGGGGCCGACGCCACCTCTCATTTGCCAACCAAACATCCAACAGCAACACCAGCAGCCAGAAGTCCACCTTGTTCATGATCAGCAACAGCAAGAGAGTCCATCTTCAACTTCCAATGTTACACCAACTATTCTAGCATTAAATCAAGGGCATCCTCCACAAAAAGCACAAGATCAGCAGCAGGAAAAATCATCATCTGCGGAGACACAAGTAGCCAGTAGCACACCTCCTCCCTCTTCATCGGTAGCTGCCAGCCGAGATAAGAAAGAGGAGATGCGGCTGCAAAAGAGAAACGAAGAAGGCTTGCACCTTCTGACCTTGCTCCTCCAATGCGCGGAGGCTGTCTCAGCTGATAATTTTGAGGAAGCTAACAAGATGCTACTTGAGATCTCGGAGCTGTCCACGCCTTTCGGAACTTCAGCGCAGCGTGTGGCTGCGTACTTCTCAGAGGCTATGTCAGCGAGGCTGGTCAGCTCATGCTTGGGGATATACGCGACGCTTCCTTCAATGCCTCAAAGCCACACCCAGAAGATGGCTTCGGCCTTTCAGGTGTTCAATGGAATTAGCCCTTTTGTCAAGTTTTCTCATTTCACAGCAAACCAAGCGATTCAAGAAGCGTTTGAAAGGGAAGAGAGAGTTCACATCATAGATCTGGACATCATGCAAGGTTTGCAGTGGCCTGGCCTATTTCATATCCTAGCATCTAGGCCCGGTGGACCACCATACGTCCGCTTGACGGGGCTGGGGACTTCCACGGAGGCTCTTGAAGCAACTGGGAAACGTTTGTCTGACTTTGCAAACAAACTGGGACTTCCCTTCGAGTTCATTCCAGTGGCCGAAAAAGTTGGAAATTTGAACCCAGAGAGGCTTAATGTTAGCAAGAGTGAGGCTGTTGCCGTCCACTGGTTGCAACACTCGCTCTACGATGTCACTGGTTCCGACACAAATATGCTTTATCTGCTGCAAAGGTTGGCACCAAAAGTAGTGACAGTGGTAGAACAGGACCTGAGCCATGCGGGATCCTTCCTGGGAAGGTTTGTGGAGGCCGTACATTACTACTCCGCACTGTTCGACTCCTTGGGAGCAAGCTACGGGGAAGAGAGCGAGGAGAGGCATGTAGTGGAACAGCAGTTGCTTTCGAGAGAGATACGCAATGTATTGGCTGTGGGAGGGCCTTCCAGAAGTGGGGATGTCAAGTTCCACAACTGGAGGGAAAAGCTTCAACAGTCTGGGTTCAAATGCATCTCTCTTGCTGGAAATGCTGCTAACCAGGCCAATCTGCTACTTGGCATGTTTCCCTCTGATGGTTATACGTTAGCAGAGGATAAGGGCACCCTCAAGCTTGGGTGGAAAGACCTTTGCTTGCTCACTGCTTCAGCCTGGAGACCTTTCCATATGAGCATTGAAACTGCTACCACCACCATCAACCACCACCACCACCACCATCGCTTTGTCACAGTCTAG

>LOC105142364

ATGGAATCGCACCAGTATTTTGGATACGGTGTCACTGGTGCGGGCCTATCCTACTCATCTTCTTATCCGTCTGTTCCTTCTATACCTAATAGGTTGTTTGGCTCATTGAAATTTGATATAGGAAATTCACCCAGCTCACCCTTCTATACTCAATTTGAGTGTGATACATACACTGCTACATTAAGTGACAGTCAGGAGTGCTACAGCTCCACAGATAATCTCTCAGGTGTTAGTCCTTCTCGTAACTCTTCTCTTGAATCCAACAGTTATTTTAATAGGCCAAGCCCTTCTGTGGACTGTAGACTAGAAAGTCTACAGCTCTTTTCTGGTGGCACTTCGTCGTTGCAAGATGCAAGTTCTAGCCAGAACATAAAACACGCGTTGCAGGAATTGGAGACTGCTCTTATGGGGCCAGATGATGACGAAGATCTGAACACGTCAAATGCTTCTCTGGGGGAAAGTAGCAGGCCACAGACATCAGATCAGAAACCTAGGGCCTGGCGCCAGGGTTCTCATGTTATTCAGAATCAGACATCTTTTGTTTCAAGGCAGAGGCAGTTGGGTGAAGGTGCTCATGTCGAGAAACGCCAAAAGTCAATGGAAGAAGTGCCTTTGCATGGTATTCCACCTGGGGATCTGAAGCAGCTGTTGATTGCATGCGCTAAAGCTCTTGCCGAAAACAACGTGAGTGCTTTTGATAAGTTGACTGAGAATGCCAGAAGTGTGGTGTCAATCAGTGGGGAACCAATCCAGCGTCTTGGTGCATACTTGATTGAGGGATTGGTAGCAAGGAAGGAGTCATCAGGTGCTAATATTTATCGTACCCTTAAGTGCAGGGAGCCTGAAGGCAAGGACTTGCTGTCTTACATGCACATCTTGTACGAAATCTGTCCATACCTGAAGTTTGGTTACATGGCAGCCAATGGAGCCATTGCTGAAGCATGCAGAAATGAGGACCGCATCCATATAATAGACTTCCAAATTGCTCAGGGAACACAGTGGATGACTCTCCTTCAAGCTCTTGCAGCCAGACCAGGTGGTGCTCCACATGTGCGTATTACAGGTATTGACGATCCTGTTTCTAAATATGCCCGTGGGGGTGGATTAGAGGCAGTTGCGGGACGGTTGAGTGCAATTTCTGAGAAATTTAATATCCCTGTAGAGTTTCATGGGGTACCAGTTTTTGCTCCAGACGTGACAAAGGAAATGCTTGACGTTAGGCCTGGAGAGGCTCTAGCTGTAAACTTTCCCTTGCAGCTCCACCACACTCCTGATGAAAGTGTTGATGTGAACAACCCAAGAGATGGGCTGCTTCGTATGATCAAATCATTCAATCCCAAGGTAGTCACTTTGGTGGAGCAAGAATCAAATACAAACACAACCCCTTTCGTCACTCGGTTTGTAGAAACTCTAAACTATTACTTGGCAATGTTTGAATCCATTGATGTGACGCTGCCAAGGGATCGAAAGGAGCGTATTAGTGTGGAGCAGCATTGTCTAGCCAGGGATATGGTAAATGTCATTGCATGTGAGGGGAAGGAGAGGGTGGAGCGCCACGAGCTCTTTCGCAAGTGGAAGTCTAGATTCATGATGGCAGGTTTCCAGCAATACCCTTTGAGTACTTATGTGAACTCTGTGATAAAGAGCCTGCTGAGGACTTACTCAGAGCACTATACATTGGTGGAAAAGGATGGAGCTATGCTGTTGGGATGGAAAGATAGGAATTTGATATCTGCTTCTGCCTGGTATTGA

>LOC105139456

ATGGCGGCAGGGCGGCTCGATGTGTCTAGGAGGCAGGGTGGTGGTGGTTATTTTGATAAGGTGAAGCGGGGTGGTGATCGTGGTTTGGTACGGAAAAATGGTTATCATAGTTCAACGATTGTGCCTAGTTTTGATTTTAGACGAGGTGGGCTTAGTTGTAGAGATGAAGAACAACAGTCAGGGGAACCGTGGCAATTTAATGCCTTCGGTTCCTCGAAATATGAGGAAGGTGAGATACCAGCAAATGAAGATGGGGTTCAATTGCCCGCAGAGAAGAAAAGGAAGTTTTCTCCTATTGTATGGGATGTTGAGGAGGAAAAAGCGAAGATTTCTTCAAAGAATAGAGTTGTTCAGAGGAGTAGTACTCGGGATGTAAATGTTGTTTCAGACGAGGATGTTGTGAAAAGTCCTGTCAAAGGAGGATTGGAGCTTTTGGTGGACAAGGATTGTGTGGATGGAGGATCAACTGATGGTATTGGATCTGAGTATCCAGCTCCTTTGTCTTCTTCGTTGCATCCAAAGAAAGATGAGGGTTATGACCAAGAGCAGGGACAAGTGGAGGAGGAAGAGCTTCCTGAAGCACGGAATATATCCATGTCCCGGTGGGCTTCTGATGATGATTCTCCAAGGGATACCACTTTGATTGATGATAAAGGAATGCACGGAGAAATGGTGTACAGGACAGATTTGATTAATAGAGAAGGGTTCCAGAGAGAAGTCTCAGATAGAGATGGATCAAGTTCATCTCTATTGGATGAAAGAGCTTACAGTGGCAGCTCTGTTTCTGAATATGAATTGCAAAATGATGTCATGGACATTGATGATATTAGGGATGAAAATGCCAGTGATAATGAGATGGAGCAAACCACTGGTGAAGAGCCCACAGTTACTAACCAAAGGGGGTTTAATATGCTAGAGGGTTGTAGAAGTGTGTTCGAGTATGAAAGACTCAATGAAATCAATGAAGGAACCTATGGTAAAGTGTACAAAGCCAGGGATAAGAAGACTGGGGAATTTGTGGCTTTGAAGAAAGTGAAGATAAATGTCGGAAGAGACAAATACTTGGAAGAGTATGGTTTCCCCTTGACATCTTTGAGGGAGATTAACATTCTTATGTCTTTTGATCACCCTTCAATTGTGAGAGTTAAAGAAGTTGTGATGGGGGACCTTGATAGTGTTTTCATGGTGATGGAGTACATGGAACATGATCTCAAGGGGCTGATGCAAGCGATGAAGCAGCCTTTCAGTACAAGTGAAGTCAAATGCTTAATGCTACAGCTTTTGGAAGGTGTCAAATATCTTCATGATAATTGGGTGCTTCACAGGGATTTGAAGACATCAAACCTTCTTTTGAATAACCAAGGAGAGTTGAAAGTATGTGATTTTGGGATGTCACGCCAGTATGGTAGCCCATTGAAGCCATATACCTCTCTGGTGGTTACTTTGTGGTACAGGGCACCAGAACTACTTCTTGGAGCAAAGAAATACTCTACAGCAGTAGACATGTGGTCAGTGGGTTGCATAATGGCTGAAATGTTGACCAAGGAACCCCTATTCACAGGGAAAGGTGAAATTGATCAGCTTGACAAGATCTTCAAAACTCTTGGCACACCAAACGAAACTATTTGGCCTGGGTTATCAAAATTGCCAGGGGCAAAAGCAAATTTTGTTAAGCAACCGTATAATCAGTTACGTAAGAAGTTTCCTTTCACACCTTTCACAGGATCTCCAGTTCTCTCTGACTCAGGATTTGACTTGCTAAACAGACTTCTAACTTATGACCCAGAGAAGCGAATAACAGCAGATGATGCTCTTAATCATCCCTGGTTTAATGAGGTCCCACTATCAAAATCAAAAGAATTCATGCCTACTTTCCCTCCTCAGTATGCAAAGAACAGGTAG

>LOC105108097

ATGACTGTGGATATGGCTATGGCTACTGAGACTCAGTTTCATGTTCTTGCTGTTGATGATTGCCTTATTGACAGAAAGTTGATTGAAAGGCTCCTTAAAACCTCTTCTTATCAAGTCACGGCAGTGGATTCAGGAAGCAAGGCCTTGGAGTTTTTGGGCTTGAATGGAGAAAATGAGCTGAGAGATTCAAAACCTGCCTCTGTTTCCCCTGACCCCTATCATCAGCACATTGAAATTAATATGATCATTACAGATTACTGTATGCCAGGAATGACAGGCTATGATCTTCTAAAAAAGATCAAGGAATCTAAATATTTCAAGGACATCCCTGTTGTGATCATGTCCTCAGAGAATGTCCCATCAAGAATCAACAGATGCCTAAAAGAAGGAGCTGAAGAGTTCTTCTTGAAGCCAGTTCAATTATCAGATGTCAACAAGCTTAGACCCCATCTAATGAAGGGAAGATGCAAGGAAGAAGAAGAAGATCAACCCAATAACAAGAGAAAGGGCATGGAAGAAATTGTTAACTCTCCAGATCGAACAAGAACAAGATACAATGATGGCTTGGAAGTTGTCTGA

>LOC105140873

ATGGCTTCATCCAGCTCTTACAACTCTCCCTGTGCTGCCTGCAAGTTCTTGAGGAGAAAATGCATGCCAGGCTGTATCTTTGCACCTTACTTCCCACCAGAGGAGCCTCAAAAATTTGCCAATGTTCACAAGATCTTTGGAGCAAGCAATGTGACAAAGCTCCTCAATGAGCTTCTCCCTCACCAAAGAGAGGATGCAGTGAACTCTCTTGCCTATGAAGCCGAGGCAAGAGTAAGGGACCCGGTTTATGGCTGTGTCGGTGCTATCTCCTTTCTCCAGAGACAAGTTCACAGGCTCCAGAAGGAACTTGATTCTGCCAACGCTGATCTGATTCGCTATGCCTGCAATGAAATCCCAACTGCATTGCCTGCACCACCAGGGGCAAGCTCAATTCAATCAATGGCTCCTCGTCAAAGGCCGGTTGAATATAATAATAGAAGGATGGGCAATGAAGGGGGTTACTATCAAGCTCCAGGCATGCCAATTCCTTATACTCTTCCTTGGAATGATAACCCTTCAGGGGATTCCAATGAAGGAGGAGGAGAAGGCAACATGTGA

>LOC105142556

ATGGAGATGGTTGACACAACGATCACTCTACAGCAACAGCAATCAATGCTGTCCAAGTTCAAGAGGATTTGTGTGTTTTGTGGGAGTAGTCAAGGCAAGAAGACTAGCTATCAAGTTGCTGCTATTGACCTTGGCAACGAATTGGTTTCACGGAACATTGATCTGGTCTATGGAGGAGGTAGCATAGGTTTGATGGGTCTGATTTCACAAGCTGTTCATGATGGTGGTCGTCATGTTATTGGAGTTATTCCCAAGACGCTCATGCCTCGAGAGTTAACTGGCGAAACAGTAGGGGAAGTGAAGGCAGTTGCAGATATGCATCAAAGGAAGGCAGAGATGGCTAAGCATTCTGATGCTTTTATTGCCTTACCAGGTGGTTATGGAACGCTGGAAGAGTTACTTGAAGTCATAACCTGGGCTCAACTTGGAATTCATGACAAACCGGTGGGACTGCTCAATGTTGATGGATACTACAATTCTCTGCTCTCATTTATTGACAAAGCTGTGGAAGAGGGGTTTATTAGTCCCAGTGCTCGCAACATTATTTTATCCGCTCCAACAGCAAAAGAGTTGGTGAAGAAACTGGAGGAGTATGTCCCCTGTCATGAAAGAGTTGCATCGAAGTTGAGCTGGGAAATTGAGCAGCTTGGCTACTCTCAAAACTATGATATCTCTAGGTGA

>LOC105136218

ATGGCTAATGGGGTTGAGAGAGAGCTGGCGGTGTTGTTTAGTGAAGAAGAGTTGAGAGAGATGAGTGGGGTCAAGAGAGGTGAAGAGTACATAGAAGTGACGTGTGGGTGTACCAGCCATAGATATGGCGATGCTGTTGGTAGGCTTAGGGGTTTTATCAATGGTGAACTTGAAATCACATGTGAATGCACTCCTGGTTGCGATGAAGATACGATGACTCCTGCTGCATTTGAAAAGCACTCTGGAAGAGAGACAGCTAGAAAATGGAAGAACAATGTCTGGGTCATAGTTAATGGGGAGAAGGTTCCATTGTCAAAGACTGTGTTGCTCAAATACTACAATCAGGCCTCAAAAAATGGCAATGGATCCCACAGGTCAAATAATGGACGAGTTTGTCACCGTGATGAGTTTGTTCGCTGTAGTGAATGTAATAAGGAACGCAGGTTCCGATTACGGACCAAAGAGGAGTGTCAGATTCACCATGATGCTTTGGCTGATGCGAACTGGAAATGTGCTGATATGCCTTTTGACAAAATAACATGTGATGATGATGAAGAACGAGCAAGTCGAAGGGTATACAGAGGTTGCACCCGTTCTCCAACATGCAAGGGCTGCACTTCCTGTGTGTGCTTTGGGTGTGAGATCTGTCGTTTCCCAGATTGCAGCTGCCAAACCTGCACTGACTTCACCAGGAATGCAAAAGTTTGA

> LOC105128586

CTCAGCTCTTGTTCTGCTACTTTCTCCGCACCATAACAAATTCCCTTCTCCGACTCTCCTGGATCTTTGATGCATTCTACCTTAGAATGTTTCTTCACAAGCATAGCTGAAACCTCTCCTTCGCTTTAATATCTCACATTCCATTCTAAACAATTGTATTTCTTCCACATTCTCATACTAACATAGTTCAAAACCTAAAAAGAAATGGCGAGGCCACGCAGCAGCAAGCGTAATTCCTCCTCAAGTTCTTACACATCAACGATAACCACCATAGCCTTCATAGCATTATGTGTTATTGGTGTTTGGATGCTAAATTCCAACTCAAAGGTTACTCCACAAACCACCAATCACGCCACCAAATCCACCACCACCAACATTGCCGCAGACGTGGATGTTTCTTCCTCCACTGAGGTAGAAAACACTGAATCCATTAACAAAAAGGAGACGCCTATTTACGAAGACAATCCAGGAGATCTTCCTGATGATGCTATCAAATCCGATGAGCCTAAAAGCAATAATGACAATGACAATAAAGAAGAAAGTAAGGACGGAAAACAAATAGATGATGGTGATAGTAAGGCTGATCAAGAGAGCTCATCACAGGATTTAAAGGGAGAAGGATCTGGCGAGGAGCAGCAGCAACAGGAAGAAAGACAAAATCAGATATCTGAAGAAAGTTCAGACAATCAAAACCAAACAGCTGATCAAACCACCCAAGAAAGTTCTCAATCTGAAGGTAGCCAGGAAACAGACGCTAATCAAGAACAAGAAACAAACGCTAATCAAGAACAAGAGCAAATTACAGTACCTGAAACTGATGATAGTAATTCAGAGGATTCTACAATTCAAAATGAAGGACAAGATCAGAAGCGGCAACAACTACAACAACAACATGTTGCAAACAGCAGTAAAGATTTACAGGATTCTCAGAACCCAGAATCAAAAAAAGACCAACAACAAGAAGAGAGTACCGGACGCAATGAAAACGATCAAGAATCTAATCAAAATGAGAAATCATATGAAGACCAACAACAACGACAACAACAAGAGAATACTGGACTCATTGAAAACACTCAAGACTCTAATCAAAATGAGAAATCATATGAAGAGCAACAACAAGAGCAAAGACAAGAAGATGCTGGAGTTCAGAATTCTTCTCAAGAATCCCAGAAGGAGGTATCTGAAGAAGATCAAAAAAAGAGAATGCAGCAGCAGCAGCAACAACAAACATCACATCATCAAGAAGCTGAAAAAGAATCCCAGGTTGATGGCAACACAAACCAAGAAACCAAGCAGGACTCAAGCTCTGGCGAGTCAGCATTTCCAGGTAGCGAGAACACAGGAATACCAAAAGAATCAAAGGAGTCAAAGAAGTCATGGTCAACTCAAGCAGCAGAGTCAGAGAATCAAAAGGAGAGAAGGAAGGAGGAATCGGACGGTAATGATAGTATGTATGGGTACACATGGCAGCTCTGTAATGTCACTGCAGGTCCTGACTATATACCTTGTTTGGATAACGAAAAGGCCTTAAGACAATTACATACAACCGGGCACTTTGAGCATAGAGAGAGACATTGTCCTGAGGTTGGACCCACTTGTTTGGTCCCACTTTCTGAAGGGTACAAAAGACCCATAACATGGCCTCAAAGTAGAGACAAGATATGGTATCATAATGTACCTCATACAAAATTGGCAGAGGTCAAGGGACATCAAAACTGGATTAAGGTTACTGGGGAGTTCTTGACCTTCCCTGGTGGTGGAACTCAGTTCATACATGGAGCTCTTCACTACATTGATTTTGTTCAACAGGCAGTGCCTAAAATTAAATGGGGAAAACACACTCGTGTGATACTGGATGTTGGGTGTGGAGTTGCAAGCTTTGGTGGTTATATTTTTGAAAGGGATGTTCTTACAATGTCATTTGCACCCAAGGATGAACATGAAGCTCAAGTTCAATTTGCCCTTGAAAGAGGAATACCTGCCATATCTGCTGTCATGGGTTCTCAGCGTCTCCCATTCCCTAGTAGGGTCTTCGATCTCATCCACTGTGCACGTTGTAGAGTCCCTTGGCACGCAGAAGGTGGCAAGCTGCTTTTGGAATTGAATCGCGTTCTCCGACCTGGAGGTTACTTTGTGTGGTCAGCAACTCCTGTTTACCAGAAGCTTCCAGAAGATGTGGAGATATGGCAAGCTATGTCTGCATTGACGGCATCTATGTGTTGGGAGCTTGTGACTATCCAGAACGACAGACTTAACGGCATTGGTGCTGCCATCTACCGCAAACCTACCACAAATAATTGCTATGATCAGAGAAAGAAAAACAGTCCCCCAATGTGTAAAAGTGACGACGACGCAAATGCTGCCTGGTATGTACCTCTGCAGGCATGCATGCACCGGATGCCTGTTTCTAAAACTGAAAGGGGGGCTAAATGGCCAGAGGACTGGCCTCAAAGACTACAAACACCTCCTTATTGGCTAAACAGCTCCCAGATGGGGATCTATGGTAAGCCAGCTCCTCAAGATTTTGCTACAGATTATGAACATTGGAAGCATGTGGTGAGCAACTCATACATGAAGGCATTGGGTATCAGCTGGTCGAATGTGAGAAATGTAATGGACATGAGAGCTGTTTATGGAGGGTTTGCAGCAGCTCTCAAGGACATGAAGATCTGGGTGTTCAATGTCGTGAACACAGACTCCCCAGATACTCTTCCGATAATATATGAGCGAGGTCTTTTTGGGATATACCATGATTGGTGTGAATCCTTCAGCTCATACCCTCGAACTTACGACCTTCTACATGCTGATCATCTCTTCTCAAAGCTGAAAAAGAGGTGCCAACTTGCTCCTCTATTGGCAGAGGTTGATAGAATCGTGAGACCTGGAGGTAAATTGATTGTTCGAGATGAGTCAAGCGCAATCGGGGAAGTTGAGAACTTGTTGAAGTCTCTGCATTGGGAGGTTCATCTAACCTTCTCCAAAGACCAAGAAGGTTTACTGAGCGCACAAAAGGGCGACTGGCGACCACAGACATATGCAGCTCTCTCCTGATCAAGAAGAGAAACAGCATCTGGCTCGTAATCTTGTGCTGCCCCTTCTTAACAGTTTATGGACTTGGCAGCTTACTGTAGAACTTTTCTTTCTTTAAAATGTAAAATCTTCTTTTGAAATACAGCTAGGTTAATGTATTTTTTATTGTCAAGTAAACCTATGAGTACTATAATCACTGCATAGAATTAAAGTATCCCGAGGGGAG

>LOC105142505

CATCAATACTCCAATTTCTCTTGAGAAACTCAAGAAAACTTCACTTCTGTCCACCATCCACTCATCCAAGATATAGAGCCTCGAGCCTCACGCCCGTCCCCATACCATTTTATAAAGTAAAACCACTTCCTGTTTCCTGTTTTACAAATCCGCTACTTGTCCTTCTATAAAATAACACACCCACGCCTGCTTCAAAACACTAGACGAAGCTTGATATATTTCTCTCACTTCAATCTGTAATTTCTGTTCCCCCAATATATCGGAAGACCCCAGAAATGAGTTATTTGAACCGGGTTTGGATGGCAGCTACCGTGGCTGCAGTTGGATACCCTGATCAAGGGTGGAAATCCAGCTTAAAATCACTGCACCATGGCAAGAGGAGGGTGTTCTCTGGCGGAGATGTGGTGGAGATCCGGCCACTTGCTGCTGCTTCTGTCGGATCGGATTGTATTGGTCTTGGAGGTTGTGGATCGGAAGGGGGAGTGAGGCAGAACGATGAGTCTCTCCGACAAGTCATGTACTTGAATTGCTGGGGCCAGGGGTAACCAGGGTGAGAAGGTCGCGGTGACCGAGTGACTCAGTGATGAGTTTCAGAGTCCAAGGTGATTGACTAGGTGGTGTTCTTTAGCCGATCAACTCAGCGGTGTGTTGGCCTGCAGCCTGGGGGATTTGGCCAGAGTTGACCAGATAGTGGATTGTGATCGATTGACCCAGCAGTGAGTTGTAGAGTCCGAGGCAAGTGACCAGGTGGTGAGTTGTGGAATCAACGTTGAGAGGGGATTGACTTTACATTGTGAATTTATTGCGGGGATGAGGAGATGATGGAAAAAAAATTAATGTGAATTTGTACAGAAACAAAATGTTAAAAATAATATTGCACTGTTTTTGTCTTA

Actin

CCAGAAGTCCTCTTCCAGCCATCTCTCATCGGAATGGAAGCTGCTGGCATCCACGAGACTACATACAACTCAATCATGAAGTGTGATGTGGATATTAGAAAGGATCTGTATGGTAACATTGTGCTCAGTGGTGGTTCCACCATGTTCCCTGGTATTGCTGACCGAATGAGCAAGGAGATCACTGCTCTTGCCCCAAGCAGCATGAAGATCAAGGTGGTTGCACCACCAGAGAGAAAATACAGTGTCTGGATTGGAGG
