# Supplementary material for: Identification of Shoot Differentiation-Related Genes in Populus euphratica Oliv
Source: Genes (Basel). 2019 Dec 11;10(12):1034. doi: 10.3390/genes10121034 (PMC6947848; doi:10.3390/genes10121034)
Supplement: Supplementary file 1 [file genes-10-01034-s001.zip › Supplementary Materials 1/Table S1 Primers for qRT-PCR Analysis.docx]

**Table S1.** Primers for qRT-PCR analysis

| **Accession No.** | **Annotation** | **Primer set** | |
| --- | --- | --- | --- |
|  |  | **Forward primer (5’-3’)** | **Reverse primer (5’-3’)** |
| LOC105115136 | AP2-like ethylene-responsive transcription factor ANT (*ANT*) | CCATATAGGCAGCAAGACCAAC | GGCGAGGAATAGTGTCTAGCAA |
| LOC105112164 | NAC domain-containing protein 90 (*CUC*) | ACACAAGAACAAGAACAAGTATGG | TCGTAAATGCTGATGTCTGGAATA |
| LOC105121805 | protein SCARECROW-like (*SCR1*) | TGCTCCACAGTTCCACCAA | CCTCCTTATTCCGTCTCCTCTC |
| LOC105142364 | PREDICTED: scarecrow-like protein 21 (*SCR2*) | GGAATTGGAGACTGCTCTTATGG | CAACTGCCTCTGCCTTGAA |
| LOC105139456 | PREDICTED: cyclin-dependent kinase 10-like (*CDK*) | GGTGATGGAGTACATGGAACAT | GGTTTGATGTCTTCAAATCCCTG |
| LOC105108097 | A-type response regulator (*ARR*) | AGAGTTCTTCTTGAAGCCAGTT | GCCATCATTGTATCTTGTTCTTGT |
| LOC105140873 | Lateral organ boundaries domain family protein (*LBD*) | GCTGTGTCGGTGCTATCTC | GACGAGGAGCCATTGATTGAA |
| LOC105142556 | cytokinin riboside 5'-monophosphate phosphoribohydrolase LOG3 (*LOG*) | GCTCAACTTGGAATTCATGACA | CTAATAAACCCCTCTTCCACAG |
| LOC105136218 | protein ULTRAPETALA 1-like (*ULT1*) | CGGTGTTGTTTAGTGAAGAAGAGT | GCAGCAGGAGTCATCGTATC |
| LOC105128586 | dehydration responsive element binding protein (*DREB*) | CTCGAACTTACGACCTTCTACAT | GCGCTTGACTCATCTCGAAC |
| LOC105142505 | wound-responsive family protein (*WIND*) | GCTGGGGCCAGGGGTAACCAGG | GGCCAAATCCCCCAGGCTGCAG |
| Previously identified | Actin | GTCCTCTTCCAGCCATCTC | TTCGGTCAGCAATACCAGG |
